# Evidence-Based Approach to the Surgical Management of Acute Pancreatitis

**DOI:** 10.1055/s-0042-1758229

**Published:** 2022-11-22

**Authors:** Alex James Sagar, Majid Khan, Niteen Tapuria

**Affiliations:** 1 Nuffield Department of Surgical Sciences, Oxford University, Oxford, United Kingdom; 2 Acute Care Common Stem, Whipps Cross Hospital, London, United Kingdom; 3 Department of General Surgery, Milton Keynes University Hospital, Milton Keynes, United Kingdom

**Keywords:** acute pancreatitis, necrotizing pancreatitis, pseudocyst, biliary pancreatitis

## Abstract

**Background** Acute pancreatitis is a significant challenge to health services. Remarkable progress has been made in the last decade in optimizing its management.

**Methods** This review is a comprehensive assessment of 7 guidelines employed in current clinical practice with an appraisal of the underlying evidence, including 15 meta-analyses/systematic reviews, 16 randomized controlled trials, and 31 cohort studies.

**Results** Key tenets of early management of acute pancreatitis include severity stratification based on the degree of organ failure and early goal-directed fluid resuscitation. Rigorous determination of etiology reduces the risk of recurrence. Early enteral nutrition and consideration of epidural analgesia have been pioneered in recent years with promising results. Indications for invasive intervention are becoming increasingly refined. The definitive indications for endoscopic retrograde cholangiopancreatography in acute pancreatitis are associated with cholangitis and common bile duct obstruction. The role of open surgical necrosectomy has diminished with the development of a minimally invasive step-up necrosectomy protocol. Increasing use of endoscopic ultrasound–guided intervention in the management of pancreatic necrosis has helped reduce pancreatic fistula rates and hospital stay.

**Conclusion** The optimal approach to surgical management of complicated pancreatitis depends on patient physiology and disease anatomy, in addition to the available resources and expertise. This is best achieved with a multidisciplinary approach. This review provides a distillation of the recommendations of clinical guidelines and critical discussion of the evidence that informs them and presents an algorithmic approach to key areas of patient management.

Acute pancreatitis is a common cause of acute abdominal pain with an incidence of 56 cases per 100,000 people per year in the United Kingdom. 1 The majority of patients experience relatively mild disease, with inflammation limited to the pancreas and recovery within a few days. However, 20% of patients will run a severe course, with an associated 13 to 35% mortality. 2 3 More severe forms of the disease may result in local complications (e.g., pancreatic necrosis) or systemic dysfunction and organ failure.

Recent advances in the management of acute pancreatitis have prompted several advisory bodies to publish guidelines to facilitate decision-making. These organizations include the following: International Association of Pancreatology (IAP)/American Pancreatology Association (APA), American Gastroenterology Association (AGA), American Society of Gastrointestinal Endoscopy (ASGE), National Institute for Health and Care Excellence (NICE), and World Society of Emergency Surgeons (WSES).

The management of acute pancreatitis has benefitted from considerable research effort. While there may still be no clear therapeutic agent to treat acute pancreatitis, 4 improvements in diagnostic protocols have allowed more definitive determination of etiology. This opens therapeutic avenues in risk reduction. 5

Evidence supports the benefit of early goal-directed resuscitation with Ringer's lactate. 6 7 8 9 Current trials are interrogating the prospect of an increasing role of epidural analgesia in pain management. 10 There has been a proliferation in potential tools for severity prediction; however, no single system of choice 11 has emerged beyond assessment for the presence and duration of organ failure. 12 Randomized controlled trials (RCTs) have provided convincing evidence for the benefit of early enteral feeding in acute pancreatitis, 13 ending the bowel rest mantra of the past.

A large meta-analysis of RCTs suggested the benefit of early endoscopic retrograde cholangiopancreatography (ERCP) in biliary pancreatitis with common bile duct (CBD) obstruction. 14 Optimal timing for this has not been determined and would be attractive for future study. Emphasis on index admission cholecystectomy has emerged from recent studies, 15 although the logistics of this in practice can result in strain on health care services.

No single technique of necrosectomy is perfect. A minimally invasive step-up approach with consideration of a combination of approaches facilitates best practice. 16 17 18 This should occur in the context of a multidisciplinary team, with the optimal approach being dependent upon patient and hospital factors.

This review considers the studies that have informed the current treatment paradigm in acute pancreatitis and suggests an algorithmic approach to key areas in the management of this disease. The focus is on areas of management most relevant to the surgical team.

## Etiology

Determination of the etiology of a patient's presentation with acute pancreatitis allows for early intervention and risk factor modification to reduce the chance of recurrence and progression to chronic pancreatitis. 19 Thorough investigation reduces the number of patients incorrectly labeled with idiopathic pancreatitis.

The most common causes of acute pancreatitis are gallstones and alcohol abuse. The exact proportion attributed to each is dependent on local epidemiology 20 ; a recent study in southern England found gallstones to account for 54% of cases, with alcohol being responsible for only 10%. 21 Twenty-three percent of cases in this group were labeled idiopathic.

Gallstones may precipitate acute pancreatitis as they migrate from gallbladder to duodenum, causing transient biliary obstruction in the process. 22

Excessive alcohol intake is the second most common cause, with the contribution to the total number of cases being a function of local alcohol practice. Perhaps, paradoxically, in the absence of concomitant smoking, moderate alcohol intake (less than 40 g/day) appears to be protective in women. 23

Hypertriglyceridemia is the next leading cause. 24 Serum triglyceride > 11.3 mmol/L indicates this as the etiology. 25 The clinical course of patients with pancreatitis and hypertriglyceridemia is more likely to be severe. 26 Although case reports exist for statins causing pancreatitis, a retrospective cohort study of almost 4 million patients found that both simvastatin and atorvastatin reduce the rate of acute pancreatitis. 27 Short case series have described the use of unfractionated heparin or insulin infusions in the acute phase. The former may release lipoprotein lipase (LPL) from endothelial cells, while the latter increases LPL synthesis in adipose and muscle cells. Although frequently used, randomized controlled data are lacking, outside of insulin infusion otherwise indicated for diabetes mellitus.

Plasmapheresis has also been trialed in hypertriglyceridemia-related pancreatitis. However, a mortality benefit could not be demonstrated over conservative management. The American Society for Apheresis states that the optimum role for apheresis therapy in acute pancreatitis is not yet established and it should be approached on a case-by-case basis. 24

Smoking is an independent risk factor for pancreatitis, with an additive effect in association with alcohol. 28 Patients with pancreatitis should be given support in alcohol and smoking cessation where appropriate to reduce recurrence rates and prevent progression to chronic pancreatitis. 1 The AGA advises that alcohol cessation support should involve brief alcohol counselling during admission. 29

Drugs account for less than 5% of cases. A significant number of drugs have been associated with acute pancreatitis. Those with the strongest association are listed in Table 1 . 22 This should be taken into account when assessing the risk–benefit profile of using these medications in susceptible individuals.

**Table 1:** Drugs strongly associated with acute pancreatitis

Drugs
Azathioprine
6-Mercaptopurine
Didanosine
Valproic acid
Angiotensin-converting enzyme inhibitors
Mesalamine

Retrospective cohort studies suggest that patients with type 2 diabetes are at increased risk of acute pancreatitis. 30 The relationship may be bidirectional: a meta-analysis demonstrated new-onset prediabetes in almost 40% of patients postpancreatitis. The relative risk of developing diabetes within 5 years of pancreatitis was 2.7. 31 Until the natural history of postpancreatitis diabetes is better understood, a defined screening protocol will not be possible; however, clinicians should be mindful of this association.

In addition to the factors mentioned, an array of more esoteric etiologies exists. The NICE advises consideration of the following: hypercalcemia, microlithiasis, hereditary causes, autoimmune pancreatitis, ampullary or pancreatic tumors, and anatomical anomalies (e.g., pancreas divisum). 1 In idiopathic pancreatitis, the WSES guidelines advise that, following recovery from the acute phase, two transabdominal ultrasounds should be performed followed by endoscopic ultrasound (EUS) and/or magnetic resonance cholangiopancreatography (MRCP) to exclude biliary etiology. 25 Secretin-stimulated MRCP may be useful if sphincter of Oddi dysfunction is suspected. 32 The IAP advises negative routine work-up should be followed by EUS and subsequently secretin-stimulated MRCP if negative. 33

A Dutch study found that following initial diagnostic work-up, 11.7% of patients were diagnosed with presumed idiopathic acute pancreatitis. This work-up involved personal history, family history, physical examination, laboratory tests, and transabdominal ultrasound scan. Further investigation, which variably involved computed tomography (CT), EUS, magnetic resonance imaging (MRI)/MRCP, repeat ultrasound, Immunoglobulin G4, and ERCP, identified a cause in 36% of patients. In those with an identified etiology, the recurrence rate was reduced. 5 Table 2 describes the approximate frequency and diagnostic clues to the less common causes of acute pancreatitis.

**Table 2:** Rare causes of acute pancreatitis, with associated approximate frequency and diagnosis

Cause	Frequency	Diagnosis
Hypertriglyceridemia	2–5%	Fasting triglycerides > 11.3 mmol/L
Drugs	5%	See Table 1 , may be rarely associated with drug reaction
Autoimmune	<1%	Type 1: elevated IgG4. Also affects salivary glands and kidneys. Type 2: occurs in younger patients. Pancreas-specific. Both respond to glucocorticoids
Trauma	<1%	History of trauma; most likely to affect midbody of pancreas
Infection	<1%	Viruses: CMV, mumps, EBV. Parasites: *Ascaris* , *Clonorchis*
ERCP	5–10% of patients undergoing ERCP	History of recent ERCP
Postsurgery	Dependent on surgery type	5–10% of patients undergoing cardiopulmonary bypass, likely due to pancreatic ischemia
Genetic	Unknown	Occurs in younger patients without alternative etiology, family history, recurrent episodes, and chronic pancreatitis
Pancreatic duct obstruction	Rare	Celiac disease, Crohn's disease, malignancy pancreas divisum, sphincter of Oddi dysfunction

Abbreviations: CMV, cytomegalovirus; EBV, Epstein–Barr virus; ERCP, endoscopic retrograde cholangiopancreatography; IgG4, immunoglobulin G4.

Source: Adapted from Forsmark et al. 22

Note: Frequency will depend on local epidemiological factors.

## Initial Management of Acute Pancreatitis

### Initial Assessment

The diagnosis of acute pancreatitis may involve a combination of history, laboratory investigations, and imaging. The revised Atlanta classification recommends that the diagnosis of acute pancreatitis be made when at least two of the following conditions are met: abdominal pain consistent with acute pancreatitis; serum amylase/lipase > 3 × upper limit of normal; and/or imaging findings consistent with acute inflammation of the pancreas. The abdominal pain is typically acute-onset, persistent, severe, epigastric pain, often radiating to the back. If the diagnosis has been reached by the first two conditions, imaging is not necessary for diagnostic purposes alone. 12

Once the diagnosis has been made, initial assessment should seek to determine disease severity and likely etiology. The former facilitates early instigation of appropriate levels of care. Careful elucidation of etiology will ensure proper risk reduction measures are taken. In some cases (e.g., autoimmune), specific targeted therapy in the acute phase may be beneficial.

**Fig. 1:**
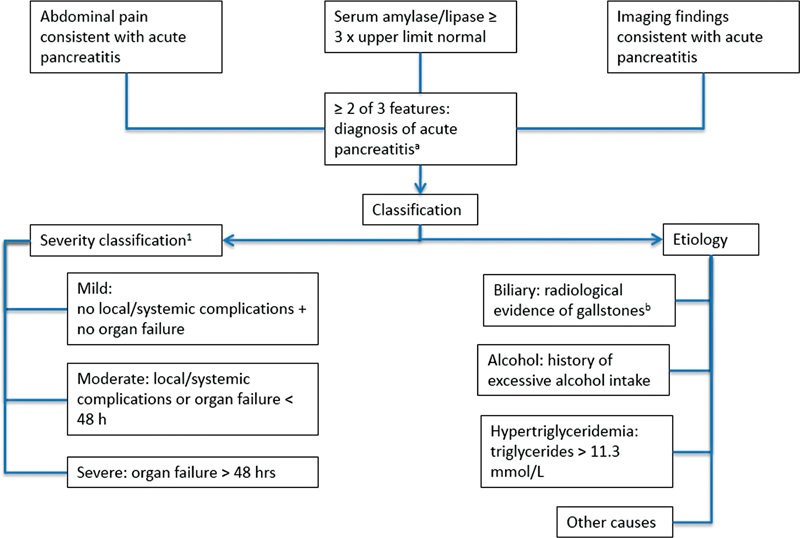
Algorithm for initial diagnosis and classification in acute pancreatitis. ^a^ Revised Atlanta classification. 12 ^b^ Alanine transaminase > 150 after 48 hours, >85% positive predictive value for biliary etiology.

Fig. 1 presents an approach to the diagnosis and classification of a patient presenting with acute pancreatitis.

### Initial imaging in Acute Pancreatitis

As mentioned, CT imaging at initial presentation may be required to reach a diagnosis in cases of diagnostic uncertainty. 25 33 Transabdominal ultrasound is undertaken shortly after index presentation to detect a biliary etiology. 25

Coexisting cholangitis, as defined by the Tokyo guidelines, 34 should be considered on admission, 33 as this impacts management. Biliary imaging may be helpful in making this diagnosis (see “Biliary Decompression in Biliary Pancreatitis”).

Management in the acute phase must focus on resuscitation, analgesia, and nutrition while ensuring that those patients who will benefit from critical care support are identified and treated in a timely manner. These pillars will be explored in the subsequent sections.

### Early Resuscitation

Systemic hypoperfusion secondary to systemic inflammatory response syndrome (SIRS) in early acute pancreatitis contributes to organ dysfunction. 12 Patients with organ dysfunction require admission to critical care 33 and continuous vital sign monitoring. 25

Systemic hypoperfusion is initially managed with careful fluid resuscitation. The AGA recommends fluid resuscitation in acute pancreatitis should be goal directed 29 : 5 to 10 mL/kg/h of Ringer's lactate should be used initially aiming for heart rate < 120/min, mean arterial pressure of 65 to 85 mm Hg, urine output of 0.5 to 1.0 mL/kg/h, and hematocrit of 35 to 44%. This approach reduced SIRS, sepsis, ventilation requirement, and mortality compared with resuscitation at rates 10 to 15mL/kg/h. 6 7

A trial of 200 patients with acute severe pancreatitis found that targeting central venous pressure of 8 to 12 mm Hg and mixed venous oxygen saturation of ≥70% reduced organ failure and mortality rate. 7 Overresuscitation with intravenous fluid is associated with acute lung injury and abdominal compartment syndrome. 25 If the patient is normovolemic with persistent hypotension, noradrenaline should be the first-line vasopressor.

In agreement with the AGA, the IAP advises Ringer's lactate as resuscitation fluid in acute pancreatitis. 33 Ringer's lactate is associated with reduced SIRS response compared with normal saline. 8 9 Some authors advise selective use of colloids for patients with acute pancreatitis and low hematocrit (<25%) or albumin (20 g/L). 35 However, this is controversial because of the association of colloids with increased mortality in patients with severe sepsis. 33

The resuscitation fluid debate remains an area of active research: a current trial continues to evaluate Ringer's lactate against normal saline (Clinicaltrials.gov NCT03642769).

## Analgesia

The management of pain in acute pancreatitis is essential. All patients should receive analgesia during their first 24 hours in hospital. Patient-controlled analgesia should be considered, 25 and intravenous analgesia will be required for severe pain. 36

Opiates are the preferred therapy, supported by Cochrane review 37 ; opiates are associated with reduced requirement for supplementary analgesia without an increase in adverse effects. The WSES advises oxycodone is preferable to morphine or fentanyl in the nonintubated patient. 25 Extrapolating from chronic pancreatitis, this may be related to its activity at the k-opioid receptor, resulting in more efficacious attenuation of visceral pain. 38 However, this is not supported by randomized controlled data. A review of nine RCTs highlights the uncertainty regarding optimal analgesic agent in acute pancreatitis. 36

Epidural analgesia should be considered in patients with severe acute pancreatitis and in those requiring intravenous opiates over an extended period. 36 Thoracic epidural analgesia may also have a therapeutic role through targeted sympathectomy, with consequent splanchnic vasodilatation and improvement in local microcirculation, as well as dampening of the inflammatory response. 39 Epidural analgesia appears safe in the critical care environment with a series of 121 patients reporting no neurologic deficit and 1 epidural abscess. 40 The potential benefits and risks in acute pancreatitis will be assessed in EPIPAN, a current multicenter RCT. 10

Nonsteroidal anti-inflammatory drugs (NSAIDs) may offer a protective effect in acute pancreatitis, 41 although further study is required to ascertain this. NSAIDs should be avoided in acute kidney injury. 25

The WSES guidelines advise adherence to the most recent perioperative acute pain management guidelines. 25 The American Society of Anesthesiologists guidelines for postoperative analgesia recommend multimodal analgesia, directed by a validated pain tool. 42

## Severity Assessment and Prediction

The traditional scoring systems for assessing acute pancreatitis (the modified Glasgow score and Ranson criteria) have largely been replaced by the revised Atlanta classification ( Fig. 1 ).

Outcome in acute pancreatitis is dictated primarily by the patient's physiological status. Transient SIRS (<48 hours) is associated with 8% mortality, while persistent SIRS (>48 hours) is associated with 25% mortality. 43 The revised Atlanta classification reliably predicts outcome. 44

The results of numerous studies of risk prediction tools have been highly variable. The WSES acknowledges that no “gold standard” prognostic score exists for predicting severe acute pancreatitis. They suggest that the Bedside Index of Severity of Acute Pancreatitis (BISAP) is probably preferred over other scoring tools. BISAP includes assessment of urea, mental status, SIRS, age, and presence of pleural effusion. 25 Advantages lie in its ability to be calculated within 24 hours of admission (compared with the 48 hours for the modified Glasgow score and Ranson criteria) and its simplicity (compared with APACHE-II and Balthazar CT severity index [CTSI]). One study found BISAP is able to predict severity, organ failure, and death as accurately as APACHE-II, while outperforming Ranson criteria. 45 By contrast, however, another study found the modified Glasgow score to have the greatest sensitivity in predicting severe episodes. 46 A meta-analysis demonstrated similar accuracy of BISAP, APACHE-II, and Ranson in predicting severity and mortality. 11

The Pancreatitis Activity Scoring System (PASS) is a novel tool that provides continuous assessment of disease activity, compared with the static prediction of severity offered by other systems. This enables tracking of patient progress through measurement of organ failure, SIRS, abdominal pain, opiate requirement, and tolerance of oral intake. 47 A prospective study confirmed its accuracy in prediction of local complications, length of stay, and severity. 48 PASS is yet to be incorporated into routine clinical practice or guideline recommendations.

Serum markers of severity include C-reactive protein (CRP) > 150 mg/L on the third day of symptoms, hematocrit > 44%, and urea > 20 mg/dL. 25 Resistin (a cytokine secreted by adipocytes) measured on day 3 of an episode of acute pancreatitis appears superior to CRP in predicting disease severity. 49

The NICE and IPA/APA guidelines recommend that patients with severe acute pancreatitis should be discussed with a specialist center. 1 33 A retrospective study using a cutoff of 118 cases per year to define high-volume centers found that patients treated at such centers experienced reduced mortality rates when adjustment was made for patient and hospital confounders. 50 However, this study looked at data from 1998 to 2006. More recent studies would be beneficial.

## Nutrition

A 2008 meta-analysis of RCTs found early nutrition support to be beneficial in acute pancreatitis, 13 with the enteral route being superior to parenteral nutrition. 51 52 A Cochrane review failed to demonstrate benefit of nasojejunal (NJ) over nasogastric (NG) feeding. 53 The latter should be the first-line enteral route should oral feeding fail.

This concept has subsequently been stratified according to the severity of an episode. In mild acute pancreatitis, a meta-analysis has demonstrated that early oral feeding is associated with reduced length of stay. 54 By contrast, in severe acute pancreatitis, enteral feeding less than 24 hours from admission is not superior to oral feeding commenced at 72 hours if tolerated. 55 Severe pancreatitis is more likely to be associated with factors such as ileus, gastric outlet obstruction, and abdominal compartment syndrome that result in failure of enteral nutrition, complicating decision-making in this group. 56

In keeping with the above-mentioned studies, the AGA advises commencing oral feeding within 24 hours if tolerated. 29 For patients with moderate-to-severe acute pancreatitis, the NICE advises that enteral feeding should commence within 72 hours. If enteral feeding fails or is contraindicated, parenteral nutrition should be started. 1 The WSES guidelines add that partial parenteral nutrition supplementation may be considered when the enteral route is unable to meet nutritional demands. 25

A practical approach is to trial early oral feeding in patients without nausea, vomiting, or abdominal distension. Where present, contraindications to enteral feeding should be sought (e.g., established ileus, intestinal obstruction), in which case parenteral nutrition will be required. Those without contraindications should be considered for either NG or NJ feeding. The latter will be necessary for patients with delayed gastric emptying, gastric outlet obstruction, and/or high aspiration risk. Patients should be closely monitored during initiation of enteral feeding. For those tolerating the feed, the rate may be increased gradually to meet nutritional requirements. Failure to tolerate will necessitate parenteral feeding, potentially as a top-up to meet requirements.

**Fig. 2:**
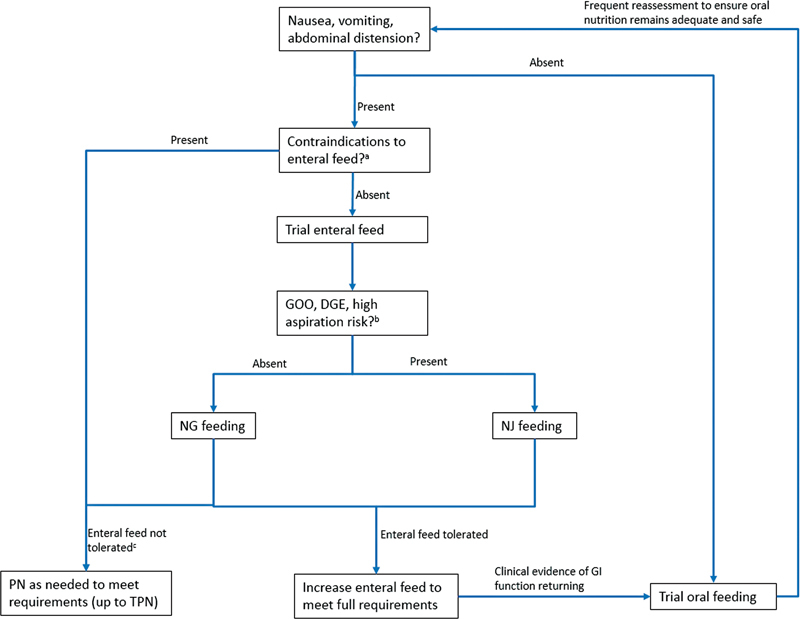
Algorithm for management of nutrition in acute severe pancreatitis. Nutrition should be addressed within 24–72 hours of presentation. DGE, delayed gastric emptying; GI, gastrointestinal; GOO, gastric outlet obstruction; NG, nasogastric; NJ, nasojejunal; PN, parenteral nutrition; TPN, total parenteral nutrition. ^a^ Contraindications include prolonged ileus, intestinal obstruction, abdominal compartment syndrome, and complex pancreatic fistula. ^b^ Consider abdominal X-ray (or CT if already planned) to assess for ileus/gastric distension/obstruction. ^c^ If failure to tolerate enteral feeding is due to tube-related complications, consideration should be given to endoscopic feeding tube insertion if patient is able to tolerate (percutaneous endoscopic gastrostomy, percutaneous endoscopic gastrostomy with jejunal extension or direct percutaneous endoscopic jejunostomy).

An algorithm for management of nutrition in severe acute pancreatitis is presented in Fig. 2 . 1 25 29 33 57

Regarding specific feeding regimens, polymeric formulas have been associated with chylous ascites and should be avoided. 58 Immunonutrition has failed to demonstrate benefit in acute pancreatitis. 59

## Antibiotic Prophylaxis

A systematic review of 10 RCTs found that prophylactic antibiotics conferred no benefit in acute pancreatitis, when studies published before 2002 were excluded. 52 The AGA advises against the use of prophylactic antibiotics, including in patients with severe pancreatitis or sterile pancreatic necrosis. This is in the NICE, WSES, and IAP/APA guidelines. 1 25 33

However, selective oral decontamination may reduce gram-negative sepsis and mortality in patients with acute severe pancreatitis. 60 By contrast, probiotics were associated with an increased mortality rate in patients with acute severe pancreatitis 61 and are not advised. 33

## Biliary Pancreatitis

### Biliary Decompression in Biliary Pancreatitis

**Fig. 3:**
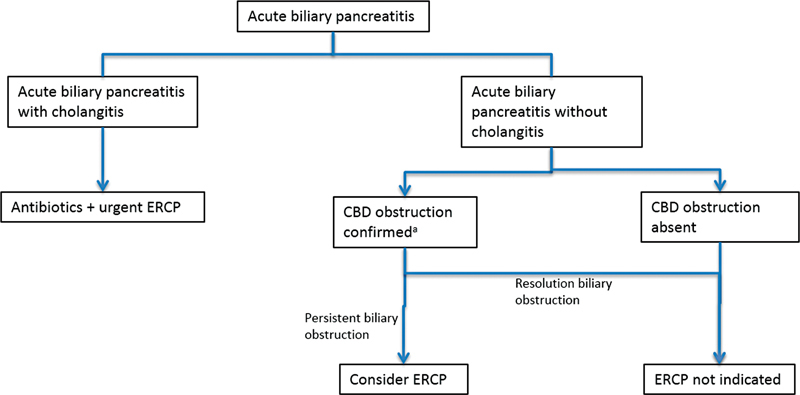
Approach to biliary decompression in acute biliary pancreatitis. CBD, common bile duct; ERCP, endoscopic retrograde cholangiopancreatography. ^a^ Magnetic resonance cholangiopancreatography/endoscopic ultrasound. (Adapted from IPA/APA, AGA, and WSES guidelines.)

An algorithm to biliary decompression in patients with acute biliary pancreatitis, based on the IPA/APA, AGA, and WSES guidelines, is presented in Fig. 3 .

A meta-analysis found that routine ERCP in patients with biliary pancreatitis is not beneficial. 14 However, for patients with associated cholangitis, early ERCP is associated with reduced mortality and complications. Similarly, for patients with pancreatitis and associated biliary obstruction, this strategy reduces local complications. A Dutch study found that patients with severe acute biliary pancreatitis and biliary obstruction benefitted from ERCP within 72 hours. 62

Accordingly, the IAP, AGA, and WSES guidelines advise against routine ERCP in acute biliary pancreatitis. Early ERCP is advised in patients with acute pancreatitis and CBD obstruction. 25 29 33 The IAP recommends that CBD obstruction be confirmed on MRCP or EUS prior to ERCP to prevent unnecessary intervention. EUS is superior in identifying small stones < 5 mm; however, it is invasive and less widely available. 33 Optimal timing for these patients is not clear 33 ; the AGA advises against ERCP < 24 hours in these cases, preferring a preceding period of resuscitation and observation. 29

For patients with acute pancreatitis and coexistent acute cholangitis, urgent ERCP is recommended within 24 hours. 25 33

For patients with biliary pancreatitis unfit for cholecystectomy, endoscopic sphincterotomy is associated with a significant risk reduction for recurrence 31 and can be performed as definitive treatment.

### Role of Cholecystectomy in Biliary Pancreatitis

The PONCHO trial demonstrated benefit of same admission over delayed (25–30 days) cholecystectomy in reducing rates of readmission for gallstone-related complications and mortality. 15 However, early cholecystectomy in patients with local collections is associated with increased infectious complications. 63

Therefore, for patients with mild biliary pancreatitis, same admission laparoscopic cholecystectomy is advised. This includes patients who have undergone ERCP and sphincterotomy at the index admission. 25 33 For patients with moderate-to-severe disease, cholecystectomy should be performed following resolution of local and/or systemic complications.

In patients labeled with presumed idiopathic pancreatitis, an RCT demonstrated that cholecystectomy reduces the rate of recurrent pancreatitis with a number needed to treat of 5, 64 implying a small but significant contingent of patients with occult gallstone disease. The decision to perform cholecystectomy in these patients should be taken on a case-by-case basis.

### Local Complications in Acute Pancreatitis

**Fig. 4:**
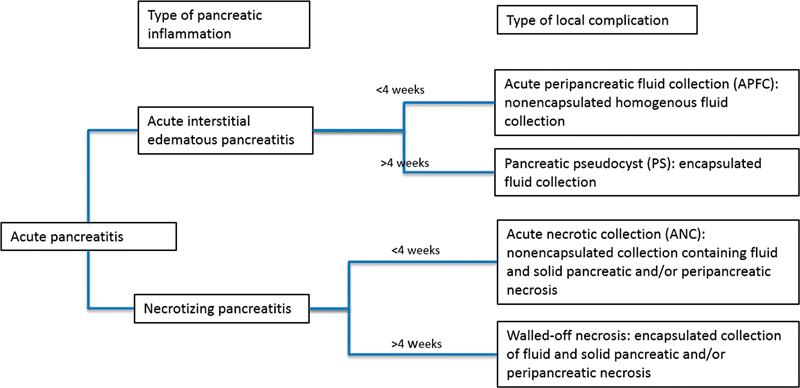
Type of pancreatic inflammation and associated local complications with definitions. 12

Acute pancreatitis may be associated with local complications. Fig. 4 illustrates the classification of acute pancreatitis according to inflammation type and the associated local complications (revised Atlanta classification 12 ).

Each of the described collections may be sterile or infected; however, infection is rare in the absence of necrosis.

Table 3 presents serum markers that can be used to identify patients at risk of local complications. 65 66 67

**Table 3:** Serum markers of local complications in acute pancreatitis 59 66 67

Markers of local complications in acute pancreatitis
Raised inflammatory markers
Blood urea nitrogen > 20 mg/dL
Packed cell volume > 44%
PCT > 3.8 ng/mL within 96 hours

Abbreviation: PCT, procalcitonin.

Patients for whom there is suspicion of local complications and those with severe acute pancreatitis should be investigated with cross-sectional imaging. Pancreas protocol CT with arterial and portal venous phases, or MRI pancreas when CT is not suitable, to assess for local complications should be delayed for 72 to 96 hours after symptom onset to improve diagnostic accuracy and yield. 25 33

The Balthazar CTSI assesses severity based on the degree of pancreatic inflammation and necrosis on initial CT. This correlates well with clinical severity; a prospective study found that CTSI calculated from imaging at a median of 6 days from symptom onset had a specificity and sensitivity for detection of moderate-to-severe disease of 97.1 and 100%, respectively. 68 A meta-analysis demonstrated that CTSI was equivalent to BISAP, Ranson, and APACHE-II in severity prediction, although less accurate than APACHE-II in mortality prediction. 11

The WSES advises a further follow-up CT 7 to 10 days following the initial CT in patients with severe acute pancreatitis. Scans in addition to these are advised in cases of failure to progress, deterioration, or when invasive intervention is required. 25

## Collections Associated with Acute Interstitial Edematous Pancreatitis

### Acute Pancreatic Fluid Collection

Up to 25% of patients with acute pancreatitis develop acute pancreatic fluid collection. Around 70 to 90% resolve without intervention. 69 A small number persist beyond 4 weeks, maturing into pseudocysts. During this time, management will focus on symptom control and nutrition support.

### Pancreatic Pseudocyst

Pancreatic pseudocysts form predominantly in the peripancreatic tissue as a result of pancreatic duct disruption.

The primary indication for intervention for a pseudocyst is symptoms: pain, weight loss, gastric outlet obstruction, and biliary obstruction. In addition, patients with asymptomatic pseudocysts should be considered for treatment if associated with pancreatic duct disruption, pressure on large vessels/diaphragm, and/or at risk of pseudocyst rupture. 1 Large size in itself is not an indication for drainage, although those larger than 6 cm tend to be symptomatic. 70

A population-based observational study found surgical drainage of pancreatic pseudocyst into the gastrointestinal (GI) tract to be superior to percutaneous drainage in terms of length of stay and mortality. 71 In turn, when anatomically feasible, endoscopic drainage is preferable to surgical drainage with reduced length of stay and improved quality of life. 70 72 The NICE recommends endoscopic drainage as the preferred treatment method, followed by surgical (open or laparoscopic) drainage into the GI tract if endoscopic methods are not suitable or successful. 1

The optimal method of drainage is dependent on the presence or absence of communication between the pseudocyst and main pancreatic duct and the anatomy of the pseudocyst. For patients with communication between cyst and pancreatic duct, endoscopic transpapillary drainage is preferable if adequate drainage can be achieved. This approach reduces the risk of perforation and bleeding while allowing identification of pancreatic duct abnormalities that may impede resolution. 70 On the contrary, for cases in which the pseudocyst is drained adequately via an endoscopic transmural approach, the addition of transpapillary stent does not improve outcome. 73

**Fig. 5:**
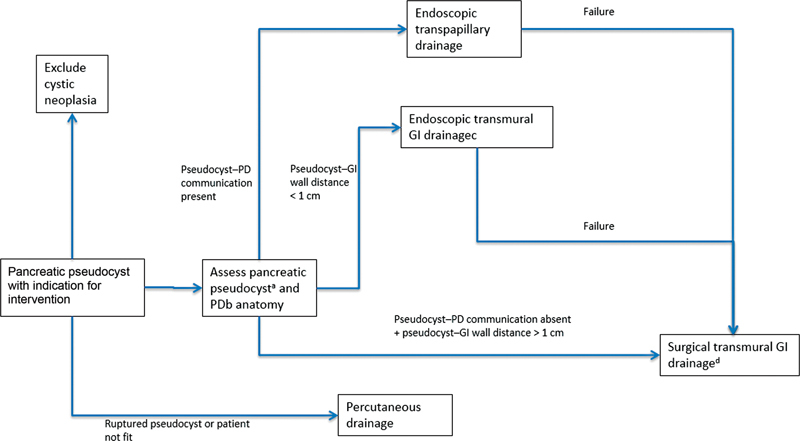
Algorithm for drainage of pseudocyst in pancreatic head, neck, or body, based on NICE, 1 ASGE, 70 and the experience of a group from West China Hospital, Sichuan, China. 74 Please see text for indications for intervention. GI, gastrointestinal; PD, pancreatic duct. ^a^ CT or transabdominal US. ^b^ MRCP or ERCP. ^c^ Endoscopic cystogastrostomy/cystoduodenostomy. ^d^ Surgical cystogastrostomy, cystoduodenostomy, or Roux-en-Y cystojejunostomy, depending on anatomical location.

An algorithm for the management of pseudocysts in the pancreatic head, neck, or body is presented in Fig. 5 . 1 70 74

Caveats exist for pseudocysts in uncinate process or tail of pancreas. Cysts in uncinate process without pseudocyst–pancreatic duct communication will require primary surgical drainage due to lack of access for endoscopic transmural drainage. Cysts in tail of pancreas cannot be drained via the transpapillary route regardless of pseudocyst–pancreatic duct communication; all will require transmural drainage (endoscopic where possible). Those in the tail of pancreas with splenic vein involvement or associated upper GI bleeding will require distal pancreatectomy and splenectomy.

## Necrotizing Pancreatitis

### Natural History of Necrotizing Pancreatitis

Around 5 to 10% of patients with severe acute pancreatitis develop necrosis. 69

For those with acute necrotic collection, 6% will become infected, 41% will resolve, and 38% will develop walled-off necrosis after 4 weeks. Of patients who develop walled-off necrosis, 21% will become infected, 59% will resolve, and 18% will persist without infection. 65 For patients with necrotizing pancreatitis, the mean time to pancreatic infection is 13.9 days. Gram-negative bacteria are the main infectious species, with *Escherichia coli* and *Pseudomonas aeruginosa* being the most common. 75

The mortality of patients with infected pancreatic necrosis without organ dysfunction is 1.4%, while that of patients with infected necrosis and organ failure is 35.2%. The mortality for patients with sterile necrosis and organ failure is 19.8%. 76

### Management of Necrotizing Pancreatitis

The presence of necrosis alone is not an indication for intervention. Indications for intervention in pancreatic necrosis are sepsis or, in sterile necrosis, symptoms, failure to thrive, or associated complications. 57

Infection of pancreatic necrosis most commonly presents in the second or third week following presentation as physiological deterioration, associated with fever, and rising inflammatory markers in the absence of another septic focus. Diagnosis is primarily by demonstration of gas in the collection on CT. Although highly specific for infection, the sensitivity of this is less impressive. 77 Some authors advocate fine-needle aspiration to confirm infection. 78 79 However, this is associated with a false-negative rate of 12 to 25% 80 and is not routine practice. 25 33 57

Intervention for pancreatic necrosis should follow a step-up management protocol. Patients who develop infection are treated initially with antibiotics. An empiric antibiotic regimen should be broad spectrum with adequate pancreatic penetration. 25 The AGA suggests carbapenems, quinolones, metronidazole, or higher-generation cephalosporins. 57 If the patient deteriorates despite antibiotics, a drain is inserted, which can be upsized if the patient fails to improve. Necrosectomy is performed if these measures are unsuccessful.

Invasive intervention (including drainage) should be delayed for at least 4 weeks from disease onset where possible, 25 33 57 70 81 with the caveat that close patient monitoring should ensure that those who are not resolving with conservative management are identified and drained promptly. 70 Necrosectomy should only be performed earlier than 4 weeks if there is a strong indication and organized collection. 57

**Fig. 6:**
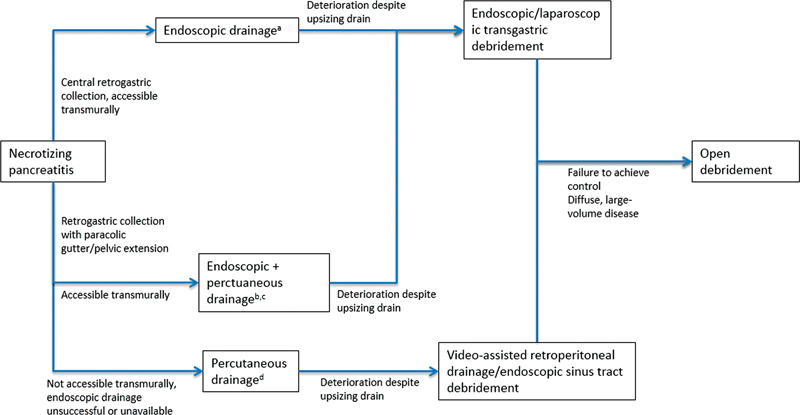
An anatomically guided step-up algorithm for management of necrotizing pancreatitis, based on AGA guideline update. Decision-making is made in the context of the multidisciplinary team, and invasive intervention should be delayed for at least 4 weeks from disease onset where possible. ^a^ Large-diameter self-expanding metal stents, in particular lumen-apposing metal stents, appear superior to plastic stents, 57 although the evidence is not yet clear. Endoscopic stenting can be combined with irrigation to improve egress of necrotic material. ^b^ The central component is drained endoscopically, with the distant collections being drained percutaneously. ^c^ Additional percutaneous drainage is often not possible for collections that extent to the right of the mesenteric vessels. ^d^ Percutaneous drainage is also required for drainage of early (<2 weeks) collections and for patients too unwell for endoscopy.

There is variability in the guidelines regarding the first-line approach for drainage. The IPA/APA and ASGE support either percutaneous or endoscopic methods. 33 70 The NICE guidelines advise for an endoscopic approach in the first instance if anatomically feasible, with the percutaneous route being reserved for those cases in which endoscopic drainage is not possible, 1 while the WSES advocate for percutaneous drainage as the first line of treatment. 25 The AGA 2020 update advises a flexible approach dependent on patient physiology, pattern of disease, the expertise of the multidisciplinary team, and available resources, with a preference for endoscopic drainage where possible. 57 The anatomical basis of decision-making recommended by the AGA is set out in Fig. 6 .

The PANTER trial supporting the step-up approach compared open necrosectomy with a step-up retroperitoneal percutaneous protocol. The latter experienced fewer complications and less multiorgan failure with similar mortality rates. 16 Percutaneous catheter drainage represented definitive management in 35% of cases. Long-term follow-up demonstrated reduced mortality, complications, pancreatic exocrine, and endocrine insufficiency in the step-up group. 82

The minimally invasive step-up approach was then extended to an endoscopic approach. The PENGUIN trial found that endoscopic transgastric necrosectomy was associated with less organ failure and fewer pancreatic fistulas compared with surgical necrosectomy. 17 The TENSION RCT 18 compared an endoscopic step-up approach with a percutaneous step-up approach. The endoscopic approach was associated with fewer pancreatic fistulas and shorter length of stay, with no difference in complication or mortality rate. EUS guidance in performing endoscopic drainage is recommended to improve safety, particularly in avoidance of bleeding complications. 57 However, this has not been subject to large RCT.

The AGA acknowledges that there remains a role for open debridement in cases not amenable to less invasive options. 57 Most trials of the step-up approach selectively enrolled patients with necrosis anatomically amenable to minimally invasive methods and used surgical necrosectomy as a first-line option rather than as part of a step-up protocol. In this context, the minimally invasive approach results in less new-onset organ failure and complications but with no significant difference in mortality on meta-analysis. 83

Importantly, if emergency surgery is needed for another indication in a patient with necrotizing pancreatitis, routine drainage or necrosectomy is not recommended unless indicated in its own right. 25

This remains an active area of clinical research. An ongoing trial is assessing standard step-up management against early drainage (Postponed or Immediate Drainage of Infected Necrotizing Pancreatitis trial). A further trial is in progress to evaluate the benefit of early percutaneous catheter drainage of sterile pancreatic fluid collections in severe acute pancreatitis.

Patients with necrotizing pancreatitis in the absence of infection are managed with supportive therapy as required, with delayed intervention as necessary for ongoing symptoms, obstructive complications, failure to thrive, or disconnected duct syndrome. 33 The ASGE advises drainage of sterile collections that remain symptomatic greater than 8 weeks from onset of disease. 70

Disconnected duct syndrome may occur as a consequence of necrotizing pancreatitis. Midbody pancreatic necrosis results in loss of the central segment of the main pancreatic duct, which causes a persistent pancreatic fistula and fluid collection. The AGA advises that good operative candidates should be managed with distal pancreatectomy, while poor candidates are best treated with endoscopic transmural drainage of the associated collection. 57

## Acute-Phase Complications

### Hemorrhage

Acute pancreatitis–associated hemorrhage most often occurs secondary to pseudoaneurysm rupture in the setting of necrotizing pancreatitis, with an associated mortality of 34 to 52%. 84

Hemodynamically stable patients should have CT angiography. Active arterial bleeds should undergo embolization, while those with venous bleeds should trial conservative management with correction of coagulopathy and octreotide infusion. Failure of these approaches will demand surgical hemostasis. Hemodynamically unstable patients will require primary surgical hemostasis, which may involve packing to gain initial control followed by embolization.

A review of 200 cases found endovascular management to be successful in 75% of cases. 85 First-line endovascular therapy is associated with reduced mortality rate compared with primary surgery.

### Venous Thrombosis

Patients with severe acute pancreatitis are at high risk for venous thromboembolic disease, particularly splanchnic venous thrombosis. There is no consensus guideline for anticoagulation management in this setting. Superior mesenteric vein or portal vein thrombosis is often managed with 6 months of therapeutic low-molecular-weight heparin with or without conversion to oral anticoagulation as appropriate, while isolated splenic vein thrombosis is treated with prophylactic low-molecular-weight heparin. 86 However, an observational study failed to show benefit of anticoagulation in rates of recanalization or mortality. 87

### Abdominal Compartment Syndrome

Abdominal compartment syndrome is defined as sustained intra-abdominal pressure > 20 mm Hg associated with new organ dysfunction. 88 Abdominal compartment syndrome in acute pancreatitis should be managed medically initially with cessation of unnecessary fluid infusion, diuretics (or ultrafiltration if necessary), methods to decrease GI tract volume (NG drainage, enemas), abdominal wall relaxation, and drainage of ascites. 89 Failure of nonoperative treatments will necessitate consideration of surgical decompression. 25 90

### Organ Failure

Early deterioration requiring critical care support is usually due to uncontrolled systemic inflammatory response leading to organ dysfunction, while late deterioration is often due to inadequately controlled sepsis. The most commonly involved systems are respiratory, renal, cardiovascular, and GI, in this order. Persistent organ dysfunction despite adequate fluid resuscitation will necessitate critical care admission. 25

### Experimental Therapies

Several potential therapeutic agents have been trialed in acute pancreatitis. Unfortunately, a systematic review of 78 RCTs failed to show consistent benefit with any therapeutic agent. 4

Examples of promising trialed agents include octreotide, which has evidence to suggest reduced rates of organ failure without improvement in mortality rates. 91 An RCT found reduced mortality with continuous regional arterial infusion of protease inhibitors with antibiotics. 92

Current trials are evaluating the potential therapeutic benefit of dabigatran (in its role as a trypsin inhibitor) and infliximab.

## Conclusion

Although the majority of cases of acute pancreatitis are mild, clinicians must be vigilant of patients developing complicated disease. Such patients present an interdisciplinary challenge that requires integrated management involving surgeons, gastroenterologists, interventional radiologists, and critical care physicians. Intervention in such cases should be carefully planned with consideration of the full range of therapeutic options. This approach improves the outcome in a complex disease.

Supplementary Material S1 **Supplementary digital content – SDC1 summary table of 45 fundamental comparative studies in the management of acute pancreatitis.**
